# Impact of *¡Míranos!* on parent-reported home-based healthy energy balance-related behaviors in low-income Latino preschool children: a clustered randomized controlled trial

**DOI:** 10.1186/s12966-023-01427-z

**Published:** 2023-03-21

**Authors:** Sarah L. Ullevig, Deborah Parra-Medina, Yuanyuan Liang, Jeffrey Howard, Erica Sosa, Vanessa M. Estrada-Coats, Vanessa Errisuriz, Shiyu Li, Zenong Yin

**Affiliations:** 1 College for Health, Community and Policy, University of Texas at San Antonio, One UTSA Circle, San Antonio, TX USA; 2 Latino Research Institute, University of Texas at Austin, 210 W. 24th Street, GWB 1.102, Austin, TX 78712 USA; 3 Department of Epidemiology and Public Health, University of Maryland School of Medicine, 660 W. Redwood Street, Baltimore, MD USA; 4 Department of Public Health, University of Texas at San Antonio, One UTSA Circle, San Antonio, TX USA; 5 School of Nursing, UT Health San Antonio, 7703 Floyd Curl Dr, San Antonio, TX USA

**Keywords:** Preschool children, Nutrition, Sleep, Screen time, Intervention

## Abstract

**Background:**

Widespread establishment of home-based healthy energy balance-related behaviors (EBRBs), like diet, physical activity, sedentary behavior, screen time, and sleep, among low-income preschool-aged children could curb the childhood obesity epidemic. We examined the effect of an 8-month multicomponent intervention on changes in EBRBs among preschool children enrolled in 12 Head Start centers.

**Methods:**

The Head Start (HS) centers were randomly assigned to one of three treatment arms: center-based intervention group (CBI), center-based plus home-based intervention group (CBI + HBI), or control. Before and following the intervention, parents of 3-year-olds enrolled in participating HS centers completed questionnaires about their child’s at-home EBRBs. Adult-facilitated physical activity (PA) was measured by an index based on questions assessing the child’s level of PA participation at home, with or facilitated by an adult. Fruit, vegetable, and added sugar intake were measured via a short food frequency questionnaire, and sleep time and screen time were measured using 7-day logs. A linear mixed effects model examined the intervention’s effect on post-intervention changes in PA, intake of fruit, vegetable, and added sugar, sleep time, and screen time from baseline to post-intervention.

**Results:**

A total of 325 parents participated in the study (CBI *n* = 101; CBI + HBI *n* = 101; and control *n* = 123). Compared to control children, CBI and CBI + HBI parents reported decreases in children’s intake of added sugar from sugar-sweetened beverages. Both CBI and CBI + HBI parents also reported smaller increases in children’s average weekday screen time relative to controls. In addition, CBI + HBI parents reported CBI + HBI parents reported increases in children’s adult-facilitated PA, fruit and vegetable intake, and daily sleep time during weekdays (excluding weekends) and the total week from baseline to post-intervention, while children in the CBI increased sleep time over the total week compared to the children in the control group.

**Conclusions:**

Parent engagement strengthened the improvement in parent-reported EBRBs at home in young children participating in an evidence-based obesity prevention program in a childcare setting. Future studies should investigate equity-related contextual factors that influence the impact of obesity prevention in health-disparity populations.

**Trial registration:**

ClinicalTrials.gov, NCT03590834. Registered July 18, 2018, https://clinicaltrials.gov/ct2/show/NCT03590834

## Background

An increasingly sedentary society has contributed to an obesogenic environment [1] that predisposes young children to dysregulation of energy balance-related behaviors (EBRBs) and an imbalance of energy intake and expenditure [2, 3]. Consequences of this imbalance include the development of obesity (i.e., excessive weight gain) and increased risk for metabolic disorders and psychosocial-behavioral problems in youth and adulthood [4, 5]. For young children ages 3–5, primary EBRBs include dietary behaviors [6, 7], physical activity (PA; [8]), sedentary behavior [9], and sleep [10]. Early childhood is a critical stage in the formulation of healthy EBRBs [8, 11], which are heavily influenced by children’s care providers (parents, family members, and childcare providers) and surrounding sociocultural, policy, and physical environments [12, 13]. For instance, young children’s food preferences and eating habits are heavily influenced by parental feeding practices [14, 15], access to healthy foods, and the food environment [12, 16]. Children from low-income minority families are disproportionally predisposed to obesogenic environments that disadvantage favorable practice of EBRBs [6], and Latino children possess higher numbers of risk factors for dysregulation of EBRBs and obesity [17, 18].

Ongoing efforts to target EBRBs to prevent obesity in childcare settings have generated mixed results in young children ages 3–5 [19–21]. For example, a recent review of 18 studies targeting PA noted there were no indications of significant PA changes in 10 of the studies [22]. In another review of 18 studies targeting healthy eating in preschool settings, 13 reported at least one positive impact on specific foods or nutrients like fruits, vegetables, and sugar [23]. Although fewer intervention studies have focused on sleep and screen time in preschool-aged children, there is recent evidence suggesting interventions may have a positive effect on sleep or screen time in children under age five [24, 25]. Similarly, reported findings from childcare-based interventions targeting the social, cultural, and physical home environments have demonstrated promising impacts on children’s body mass index (BMI) and EBRBs [26, 27]. However, a gap remains in culturally tailored intervention to target American Latino children and their families [28, 29] who are at increased risk for obesity [30].

*¡Míranos!* Look at Us, We Are Healthy! (*¡Míranos!*) is a multi-level obesity prevention intervention targeting multiple EBRBs that uses evidence-based strategies to reduce barriers and enhance enablers of obesity prevention for low-income Latino children enrolled in Head Start [31]. In this report, we examine the impact of *¡Míranos!* on parent-reported, secondary outcomes (i.e., child’s PA, screen time, sleep, and diet), following the completion of the 8-month comprehensive intervention. The primary hypothesis of the study was that compared to children in the control group, children in the center-based intervention (CBI) or the center-based plus home-based intervention (CBI + HBI) would have significantly higher levels of parent-reported PA, sleep duration, and intake of fruit and vegetables, as well as lower levels of screen time and added sugar and sugar-sweetened beverage intake at the end of the intervention.

## Methods

### Research design and intervention

This research study was a clustered randomized controlled trial conducted in 12 Head Start childcare centers from two agencies in San Antonio, Texas [32]. Head Start is a federally funded program that provides services in school readiness, health, and family support to children ages 3 to 5 from low-income families in the United States [33]. The study was powered on the primary outcome (BMI change) but not on the secondary outcomes (diet, PA, screen time, and sleep behaviors). The study sample included 12 Head Start Centers, 4 centers per group, with an average of 29 children per center (*N* = 444) at baseline to achieve 80% power to detect a group difference of 0.53 in BMI change at the end of the intervention (i.e., mean change of -0.03 in the CBI group or the CBI + HBI group vs. mean change of 0.5 in the control group) using a two-sided t-test with a significance level of 5%, an intraclass correlation of 0.003, and a standard deviation (SD) of 1.147 (PASS Version 11).

Twelve Head Start centers from two Head Start agencies were randomly assigned to one of three conditions: the combined center-based plus home-based (CBI + HBI), the center-based only intervention (CBI), or the control condition in a 1:1:1 ratio. Agency #1 had four centers and agency #2 had eight centers. Treatment randomization was stratified by Head Start agency (agency one vs. agency two) and center size (small (≤ 2, 3-years-old classrooms) vs. large (≥ 3, 3 years-old classrooms)) and generated by the study biostatistician using R version 3.3.2 (R Development Core Team, Austria). All 3-year-old children enrolled in Head Start were eligible to participate in the study. Written parent or guardian consent was obtained for each child. All study protocols were approved by the institutional review board at the University of Texas at San Antonio (IRB# 18–187). Two cohorts of participants received the 8-month intervention (cohort 1: September 2018 to May 2019; cohort 2: September 2019 to May 2022).

The rationale and development of the intervention have been described previously [32]. Briefly, ¡Míranos! included multi-level systems changes to policy, education, and reinforcement of healthy behaviors among children and their families. In the CBI, center nutrition and PA policy modifications were implemented to ensure center environments were conducive to healthy behaviors and practices. Centers increased children’s exposure to new foods and other EBRBs by modifying meal patterns to include more fruits and vegetables, employing food tastings, adding supervised PA during transition periods, and implementing health contests (e.g., drinking water, reducing TV watching). Additionally, Head Start teachers provided age-appropriate health education throughout the center day and implemented an enhanced gross motor program. Head Start staff also participated in a voluntary staff wellness program designed to develop PA- and nutrition-related health literacy and promote a healthy lifestyle. Finally, parents received monthly newsletters containing tips to help children meet PA, nutrition, screen time, and sleep time recommendations at home and food tasting recipes that mirrored center-based food tastings.

CBI + HBI parents were invited to participate in 8 monthly education sessions with information on topics related to obesity prevention recommendations and practices and strategies to develop healthy EBRBs. Specific topics are detailed elsewhere [34]. Trained Head Start parents served as peer educators and delivered education sessions using wall posters scheduled for two days of the week during child pick-up time. Parents interacted with peer educators while viewing posters and completed a scavenger hunt form. Parents that completed the scavenger hunt received a take-home bag containing materials (e.g., health-themed storybook, bilingual family activities newsletter, developmentally appropriate interactive game) that reinforced the session topic. Following each education session, parents participated in a one-week family health challenge to improve one EBRB, of their choice, at home. Additionally, Head Start family service workers offered parenting skills training and support to promote the development of healthy EBRBs during three home visits by setting family goals and developing an action plan. The parents worked with the family service worker to evaluate and refine the action plan in follow-up visits.

Head Start staff at control centers implemented an obesity prevention program endorsed by Head Start entitled “I Am Moving, I Am Learning.” Parents of children enrolled in control centers were also invited to participate in a nutrition-themed literacy education program provided by a local grocery chain.

*¡Míranos!* was implemented in preschool classrooms with 3-year-olds over an 8 months (early fall to late spring). During the program implementation period, we conducted weekly, online surveys of center directors and teachers to collect specific implementation data. Via this survey, program staff kept researchers informed on various activities completed (e.g., distribution of program newsletters, health contests, food tastings). Teachers reported data on the number of children, parents, and staff who participated in the health contests, which indicated reach. Head Start staff who conducted the home visits completed a *¡Miranos!* log to report the number of home visits and parent attendance at each educational session. Assessment of intervention fidelity indicated that 88% of the centers distributed all newsletters to parents and caregivers each week from both CBI and CBI + HBI centers. In 6 of the 8 intervention centers (67%), center directors reported that all 8 health contests were held during the year with the other 2 centers completing 4 to 5 health contests. On average, 67% of parents/caregivers from the CBI + HBI centers received all 3 home visits, with 82% of parents receiving at least 1 visit. On average, 92% of parents/caregivers from the CBI + HBI centers attended education sessions with attendance for each session ranging from 86 to 96%.

### Data collection

Sources of child demographic and health history data included Head Start center records and self-administered parent questionnaires, which were available in English and Spanish. Estimates of family income and employment were derived using the child’s home address from the 2010 US Census block-level data. Parents reported child behaviors at home, including screen time, sleep, intake of fruit, vegetables, and sugar, and PA [35].

#### Parent-Reported PA

Parents were asked 5 specific questions related to time spent with children in various activities during a typical day (i.e., played outside with me, played outside with neighborhood children, played outside with other family members, played on a sports team, walked with me). Responses included “None”, “30 Minutes”, “1 Hour”, “2 Hours”, “3 Hours”, and “ ≥ 4 Hours”. A factor score was generated to indicate the level of child’s participation in PA with facilitation and/or supervision of adults at home or in the community. This tool was used in a previous study but has not been validated [31].

#### Screen and sleep time

Parents were instructed to document their child’s hours and minutes of screen time (i.e., video games, TV watching, phone/tablet) and sleep time, including nap time, at home for each day of the past week [36]. For each child, average daily screen time or sleep time in hours was calculated over seven days (total week), per weekday (Monday through Friday), and per weekend (Saturday and Sunday). These tools have not been validated but used previously in young children [36].

#### Food intake

To assess children’s dietary intake at home, parents completed a modified version of the validated National Health and Nutrition Examination Survey (NHANES; [35]. Data were collected on 18 questions that assessed the frequency of fruits, vegetables, and sugar that children ate at home over the past month (i.e., never, 1–3 times in the last month, 1–2 per week, 3–4 per week, 5–6 times per week, 1–2 per day, 3–5 per day, or 6 or more times per day). To calculate the number of servings per day of fruit (cups), vegetables (cups), fruit and vegetables (cups), and added sugar (teaspoons), all frequencies were converted into servings per day and multiplied by standard serving sizes for age and sex [37]. The maximum allowable serving sizes set by NHANES were used for reported serving sizes that met or exceeded the maximum allowable serving size per day; this minimizes the likelihood of extreme and unrealistic serving sizes [38]. Fruits included fresh, canned, frozen fruit, and 100% fruit juices. Vegetables consisted of leafy green vegetables, potatoes, beans, tomato and tomato sauce, and other vegetables. Total added sugar included added sugar from foods such as candy, cookies, cakes, pan dulce, ice cream, or other frozen desserts and sugar-sweetened beverages, including soda, honey added to coffee or tea, and sweetened fruit drinks.

Because the COVID-19 epidemic disrupted the program implementation and data collection in Spring 2020, this analysis included only data collected from August 2018 to June 2019. The COVID-19 pandemic did not affect the data collected during this period and what is reported in this manuscript.

### Statistical analysis

We employed descriptive statistics to summarize the demographic characteristics of both Head Start Centers and study participants as well as each outcome of interest (parent-reported PA, diet, screen time, and sleep time) measured at each time point (baseline vs. post-intervention). Baseline, post-intervention, and change scores (post-intervention – baseline) for each outcome of interest were compared among the three groups (CBI, CBI + HBI, Control) using the Kruskal–Wallis H test. For each outcome of interest, we used a 3-level (time nested within child, and child nested within center) linear mixed effects model (LMM) to examine the treatment effect. Two random effects were included in the LMM, one to account for the correlation among two measures nested within the same child, and the other to account for the correlation among children nested within the same center. We assumed data were missing at random. In LMM, time (baseline vs. post-intervention), treatment group (CBI vs. CBI + HBI vs. Control), the interaction between time and treatment group, and center size (large vs. small) were fixed design-related predictors that were always kept in the final model, regardless of whether they were statistically significant.

We considered the following confounders in the full LMM for each outcome of interest: child’s age at baseline, gender, race/ethnicity, asthma, mother’s education, language spoken most often at home, parent marital status, family history of diabetes, and child’s BMI status at baseline. We employed a backward model selection to remove one non-significant (*p* > 0.05) confounder at a time from the confounder list above, and used Akaike’s information criterion (AIC) and Bayesian information criterion (BIC) to guide the model selection process to select the final reduced model. All analyses were performed using Stata/SE (version 17).

## Results

**Fig. 1:**
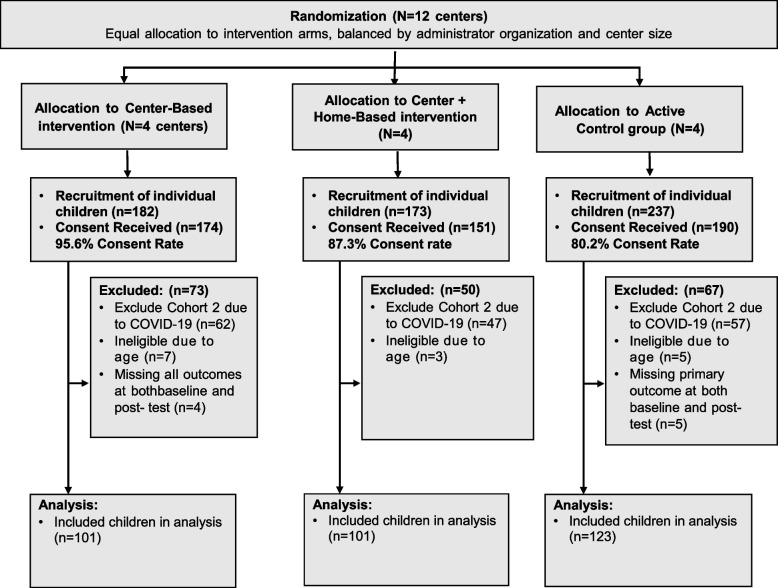
Data Flow Diagram

Overall, 325 parents participated in the study and provided data on at least one of the outcomes of interest (see Fig. 1). Of these 325 parents, 311 (88%) completed the survey at baseline, and 215 (66%) completed the post-intervention survey. Children were primarily female (57%) and Latino (87%) with a mean age of 3.59 (SD = 0.29) years at baseline. Approximately 13% of children had a diagnosis of asthma, 41% had family members with diabetes, 65% were normal weight, 15% were overweight, and 17% were obese (see Table 1). The majority of mothers reported attaining at least a high school degree, and English was the primary language spoken at home by the majority of the children. At baseline, there were no significant differences in children’s characteristics across the three conditions, with the exception that more children in the control group were enrolled in large-sized health centers compared to the other two groups.

**Table 1:** Demographics and characteristics of head start centers and study participates

Variables	H + CBI (*N* = 101)	CBI (*N* = 101)	Control (*N* = 123)	Total (*N* = 325)	P-value
**Center, n (%)**					0.02
Small	65 (64.36)	71 (70.3)	65 (52.85)	201 (61.85)	
Large	36 (35.64)	30 (29.7)	58 (47.15)	124 (38.15)	
**Child age at baseline, yr**					0.47
Median [Q1, Q3]	3.64 [3.38, 3.83]	3.55 [3.36, 3.76]	3.6 [3.31, 3.92]	3.59 [3.34, 3.84]	
Mean ± SD	3.6 ± 0.29	3.56 ± 0.26	3.6 ± 0.32	3.59 ± 0.29	
**Child sex, n (%)**					0.14
Male	44 (43.56)	36 (35.64)	60 (48.78)	140 (43.08)	
Female	57 (56.44)	65 (64.36)	63 (51.22)	185 (56.92)	
**Child race/ethnicity, n (%)**					0.15
Non-H AA	7 (6.93)	9 (8.91)	4 (3.25)	20 (6.15)	
Hispanic	83 (82.18)	86 (85.15)	113 (91.87)	282 (86.77)	
Other	11 (10.89)	6 (5.94)	6 (4.88)	23 (7.08)	
**Asthma, n (%)**	8 (7.92)	16 (15.84)	17 (13.82)	41 (12.62)	0.21
**Mother education, n (%)**					0.72
Less than a High school degree	11 (10.89)	12 (11.88)	13 (10.57)	36 (11.08)	
High School Degree/GED	46 (45.54)	39 (38.61)	58 (47.15)	143 (44)	
College or Technical School Degree	31 (30.69)	41 (40.59)	41 (33.33)	113 (34.77)	
N/A or missing	13 (12.87)	9 (8.91)	11 (8.94)	33 (10.15)	
**Language spoken most often at home, n (%)**					0.67
English	58 (57.43)	61 (60.4)	61 (49.59)	180 (55.38)	
Spanish or other	23 (22.77)	24 (23.76)	31 (25.2)	78 (24)	
English and Spanish equally	13 (12.87)	11 (10.89)	22 (17.89)	46 (14.15)	
Not reported	7 (6.93)	5 (4.95)	9 (7.32)	21 (6.46)	
**Parents married, n (%)**	38 (37.62)	36 (35.64)	46 (37.4)	120 (36.92)	0.95
**Family members with diabetes, n (%)**	44 (43.56)	44 (43.56)	45 (36.59)	133 (40.92)	0.46
**BMI status**					0.98
Underweight	3 (2.97)	4 (4)	4 (3.25)	11 (3.4)	
Normal	67 (66.34)	62 (62)	81 (65.85)	210 (64.81)	
Overweight	13 (12.87)	16 (16)	19 (15.45)	48 (14.81)	
Obese	18 (17.82)	18 (18)	19 (15.45)	55 (16.98)	

*P* values are comparing the differences among the three groups

Table 2 reports the means and standard deviations of parent-reported PA, diet, screen time, and sleep time outcomes at baseline and post-intervention. Children in the control centers had the highest amount of screen time on weekdays (2.16 h/day) and the entire week (2.19 h/day), and the lowest amount of sleep time on weekdays (9.84 h/day) and the entire week (10.0 h/day) post-intervention. Children in the control centers also had the highest intake of total added sugar (3.39 tsp/day) at post-intervention. There were no significant differences between the three groups for any outcome at baseline or change scores. There were no significant differences in any outcome between children with completed data and children with missed post-intervention surveys (data not shown).

**Table 2:** Parent-reported child physical activity, diet, screen time, and sleep outcomes at baseline, post-intervention and change scores

Variables (per day)	H + CBI (*N* = 101) M ± SD	CBI (*N* = 101) M ± SD	Control (*N* = 123) M ± SD	Total (*N* = 325) M ± SD	P-value
**Physical Activity**					
**Adult Facilitated PA**					
Baseline^1^	-0.21 ± 0.93 85	-0.24 ± 0.96	-0.08 ± 0.95	-0.17 ± 0.95	0.31
Post-Intervention^2^	0.26 ± 1.12	0.07 ± 0.93	0.07 ± 1.09	0.14 ± 1.08	0.56
Change^3^	0.39 ± 1.03	0.30 ± 1.00	0.14 ± 1.05	0.29 ± 1.03	0.51
**Diet**					
**Fruit (cups)**					
Baseline^4^	1.04 ± 0.86	1.60 ± 1.95	1.15 ± 1.07	1.24 ± 1.35	0.72
Post-Intervention^5^	1.35 ± 1.66	1.32 ± 1.24	1.39 ± 1.44	1.35 ± 1.46	0.93
Change^6^	0.42 ± 1.76	-0.17 ± 1.91	0.14 ± 1.56	0.14 ± 1.77	0.89
**Vegetables (cups)**					
Baseline^7^	0.57 ± 0.47	0.99 ± 1.67	0.64 ± 0.56	0.72 ± 1.01	1.00
Post-Intervention^8^	0.88 ± 1.34	0.73 ± 0.68	0.65 ± 0.47	0.76 ± 0.94	0.95
Change^9^	0.39 ± 1.52	-0.19 ± 1.37	0.04 ± 0.59	0.01 ± 1.28	0.85
**Fruit and Vegetables (cups)**					
Baseline^10^	1.59 ± 1.11	2.63 ± 3.28	1.84 ± 1.43	1.99 ± 2.11	0.66
Post-Intervention^11^	2.22 ± 2.69	2.07 ± 1.69	2.04 ± 1.67	2.12 ± 2.10	0.93
Change^12^	0.79 ± 2.95	-0.45 ± 2.96	0.12 ± 1.80	0.19 ± 2.71	0.75
**Added Sugar (tsp)**					
Baseline^13^	3.49 ± 4.09	3.86 ± 7.25	3.11 ± 4.15	3.45 ± 5.25	0.31
Post-Intervention^14^	2.40 ± 2.50	2.37 ± 2.06	**3.39** ± 2.71	2.66 ± 2.44	0.03*
Change^15^	-0.68 ± 3.27	-1.10 ± 5.58	-0.50 ± 5.47	-0.77 ± 4.80	0.28
**Added Sugar from food only (tsp)**					
Baseline^16^	1.59 ± 2.52	2.03 ± 4.36	1.45 ± 2.36	1.66 ± 3.12	0.32
Post-Intervention^17^	1.41 ± 1.59	1.27 ± 1.35	1.83 ± 1.63	1.48 ± 1.53	0.06^
Change^18^	-0.11 ± 2.50	-0.50 ± 3.84	-0.02 ± 3.02	-0.21 ± 3.13	0.51
**Added Sugar from beverages only (tsp)**					
Baseline^19^	1.79 ± 2.48	1.80 ± 3.26	1.71 ± 2.47	1.76 ± 2.72	0.3
Post-Intervention^20^	1.11 ± 1.64	1.09 ± 1.16	1.52 ± 1.55	1.22 ± 1.47	0.05^
Change^21^	-0.54 ± 1.59	-0.59 ± 2.45	-0.53 ± 3.45	-0.55 ± 2.51	0.33
**Screen time**					
**Weekday (hrs)**					
Baseline^22^	1.55 ± 0.90	1.55 ± 0.77	1.57 ± 0.81	1.56 ± 0.83	0.9
Post-Intervention^23^	1.55 ± 0.81	1.69 ± 0.85	2.16 ± 0.75	1.83 ± 0.84	< 0.001*
Change^24^	0.03 ± 0.84	0.19 ± 0.85	0.60 ± 0.84	0.30 ± 0.88	< 0.001*
**Weekend (hrs)**					
Baseline^25^	1.89 ± 1.20	1.86 ± 1.08	1.96 ± 0.96	1.91 ± 1.08	0.72
Post-Intervention^26^	1.68 ± 1.09	1.95 ± 1.15	2.25 ± 1.30	1.94 ± 1.19	0.06^
Change^27^	-0.29 ± 1.23	0.11 ± 0.96	0.17 ± 1.49	-0.03 ± 1.24	0.39
**Total Week (hrs)**					
Baseline^28^	1.64 ± 0.93	1.62 ± 0.78	1.67 ± 0.80	1.64 ± 0.83	0.87
Post-Intervention^29^	1.60 ± 0.82	1.76 ± 0.87	2.19 ± 0.78	1.87 ± 0.86	< 0.001*
Change^30^	-0.30 ± 0.82	0.20 ± 0.80	0.52 ± 0.85	0.25 ± 0.85	< 0.001*
**Sleep**					
**Weekday (hrs)**					
Baseline^31^	9.83 ± 0.76	9.85 ± 0.66	9.86 ± 0.76	9.84 ± 0.73	0.99
Post-Intervention^32^	10.1 ± 0.69	10.1 ± 0.79	9.84 ± 0.68	10.0 ± 0.72	0.01*
Change^33^	0.27 ± 0.76	0.20 ± 0.78	0.06 ± 0.82	0.17 ± 0.79	0.19
**Weekend (hrs)**					
Baseline^34^	10.5 ± 1.1	10.7 ± 1.21	10.9 ± 1.09	10.7 ± 1.13	0.28
Post-Intervention^35^	10.8 ± 1.25	10.8 ± 0.99	10.7 ± 1.40	10.8 ± 1.20	0.95
Change^36^	0.23 ± 1.51	0.04 ± 1.36	-0.06 ± 1.72	0.1 ± 1.51	0.94
**Total Week (hrs)**					
Baseline^37^	10.1 ± 0.76	10.1 ± 0.70	10.1 ± 0.78	10.1 ± 0.75	0.97
Post-Intervention^38^	10.3 ± 0.65	10.3 ± 0.77	10.0 ± 0.70	10.2 ± 0.72	0.002*
Change^39^	0.23 ± 0.69	0.15 ± 0.94	-0.12 ± 0.97	0.08 ± 0.86	0.05^

Entries are Mean ± SD

*P* values are comparing the differences among the three groups based on Kruskal–Wallis H test

^ 0.05 <  = *p* < 0.1

^*^ *p* < 0.05

Change = post-intervention—baseline

^1^ Sample sizes are 84, 74 and 106 in the HBI&CBI, CBI and control groups, respectively

^2^ Sample sizes are 71, 67 and 53 in the HBI&CBI, CBI and control groups, respectively

^3^ Sample sizes are 62, 52 and 47 in the HBI&CBI, CBI and control groups, respectively

^4^ Sample sizes are 90, 80 and 109 in the HBI&CBI, CBI and control groups, respectively

^5^ Sample sizes are 75, 70 and 54 in the HBI&CBI, CBI and control groups, respectively

^6^ Sample sizes are 69, 58 and 51 in the HBI&CBI, CBI and control groups, respectively

^7^ Sample sizes are 81, 73 and 99 in the HBI&CBI, CBI and control groups, respectively

^8^ Sample sizes are 72, 69 and 54 in the HBI&CBI, CBI and control groups, respectively

^9^ Sample sizes are 60, 53 and 45 in the HBI&CBI, CBI and control groups, respectively

^10^ Sample sizes are 81, 73 and 96 in the HBI&CBI, CBI and control groups, respectively

^11^ Sample sizes are 72, 68 and 54 in the HBI&CBI, CBI and control groups, respectively

^12^ Sample sizes are 60, 52 and 44in the HBI&CBI, CBI and control groups, respectively

^13^ Sample sizes are 81, 79 and 102 in the HBI&CBI, CBI and control groups, respectively

^14^ Sample sizes are 74, 71 and 54 in the HBI&CBI, CBI and control groups, respectively

^15^ Sample sizes are 62, 58 and 49 in the HBI&CBI, CBI and control groups, respectively

^16^ Sample sizes are 85, 79 and 105 in the HBI&CBI, CBI and control groups, respectively

^17^ Sample sizes are 75, 71 and 55 in the HBI&CBI, CBI and control groups, respectively

^18^ Sample sizes are 66, 58 and 51 in the HBI&CBI, CBI and control groups, respectively

^19^ Sample sizes are 87, 81 and 109 in the HBI&CBI, CBI and control groups, respectively

^20^ Sample sizes are 76, 71 and 55 in the HBI&CBI, CBI and control groups, respectively

^21^ Sample sizes are 68, 60 and 52 in the HBI&CBI, CBI and control groups, respectively

^22^ Sample sizes are 94, 97 and 118 in the HBI&CBI, CBI and control groups, respectively

^23^ Sample sizes are 82, 80 and 102 in the HBI&CBI, CBI and control groups, respectively

^24^ Sample sizes are 77, 76 and 97 in the HBI&CBI, CBI and control groups, respectively

^25^ Sample sizes are 82, 70 and 83 in the HBI&CBI, CBI and control groups, respectively

^26^ Sample sizes are 61, 59 and 52 in the HBI&CBI, CBI and control groups, respectively

^27^ Sample sizes are 55, 47 and 37 in the HBI&CBI, CBI and control groups, respectively

^28^ Sample sizes are 95, 98 and 118 in the HBI&CBI, CBI and control groups, respectively

^29^ Sample sizes are 82, 80 and 102 in the HBI&CBI, CBI and control groups, respectively

^30^ Sample sizes are 78, 77 and 97 in the HBI&CBI, CBI and control groups, respectively

^31^ Sample sizes are 96, 98 and 116 in the HBI&CBI, CBI and control groups, respectively

^32^ Sample sizes are 80, 79 and 96 in the HBI&CBI, CBI and control groups, respectively

^33^ Sample sizes are 76, 78 and 91 in the HBI&CBI, CBI and control groups, respectively

^34^ Sample sizes are 84, 70 and 89 in the HBI&CBI, CBI and control groups, respectively

^35^ Sample sizes are 65, 64 and 49 in the HBI&CBI, CBI and control groups, respectively

^36^ Sample sizes are 61, 48 and 35 in the HBI&CBI, CBI and control groups, respectively

^37^ Sample sizes are 98, 98 and 117 in the HBI&CBI, CBI and control groups, respectively

^38^ Sample sizes are 80, 79 and 96 in the HBI&CBI, CBI and control groups, respectively

^39^ Sample sizes are 78, 78 and 92 in the HBI&CBI, CBI and control groups, respectively

Findings from adjusted LMMs indicated that children in both the CBI (+ 0.31 [95% CI: 0.05, 0.57], *p* = 0.02) and the CBI + HBI (+ 0.44 [0.20, 0.68], *p* = 0.0001) groups significantly increased the index score of adult-facilitated PA participation from baseline to post-intervention while a small but not significant increase was found in control children (+ 0.14 [-0.12, 0.41], *p* = 0.29). There were no significant between-group differences in the change of adult-facilitated PA between CBI and control and CBI + HBI and control, although the trend favored both intervention groups (Table 3).

**Table 3:** Treatment effects on parent-reported change in child physical activity, diet, screen time, and sleep outcomes.^1^

Outcomes	H + CBI (*n* = 101)	CBI (*n* = 101)	Control (*n* = 123)	Difference (H + CBI – Control)			Difference (CBI – Control)	
	**Mean change (SE)**	**Mean change (SE)**	**Mean change (SE)**	**Difference [95% CI]**	***P* value**	**Difference [95% CI]**		***P* value**
**Physical Activity**								
Adult Facilitated PA^2^	0.44 (0.12)*	0.31 (0.13)*	0.14 (0.14)	0.30 [-0.06, 0.66]	0.11	0.17 [-0.20, 0.54]		0.38
**Diet**								
Fruit (cups)^3^	0.35 (0.19)^	-0.26 (0.20)	0.20 (0.21)	0.15 [-0.40, 0.71]	0.59	-0.46 [-1.02, 0.11]		0.12
Vegetable (cups)^4^	0.31 (0.15)*	-0.26 (0.16)	0.03 (0.17)	0.28 [-0.16, 0.73]	0.21	-0.29 [-0.74, 0.17]		0.22
Fruit and Vegetable (cups)^5^	0.67 (0.31)*	-0.56 (0.32)^	0.15 (0.33)	0.52 [-0.37, 1.41]	0.25	-0.70 [-1.61, 0.21]		0.13
Added Sugar Total (tsp)^6^	-0.89 (0.59)	-1.37 (0.60)*	0.18 (0.64)	-1.08 [-2.78, 0.63]	0.22	-1.56 [-3.28, 0.16]		0.08^
Added Sugar from food (tsp)^7^	-0.17 (0.36)	-0.73 (0.38)^	0.31 (0.39)	-0.48 [-1.53, 0.57]	0.37	-1.04 [-2.11, 0.04]		0.06^
Added Sugar from Sugar Sweetened Beverages (tsp)^8^	-0.60 (0.29)*	-0.67 (0.31)*	-0.26 (0.32)	-0.34 [-1.19, 0.52]	0.44	-0.41 [-1.27, 0.46]		0.36
**Screen Time**								
Weekday (hrs)^9^	0.02 (0.09)	0.16 (0.09)^	0.59 (0.08)*	-0.57 [-0.82, -0.33]	< 0.001*	-0.43 [-0.68, -0.18]		0.006*
Weekend (hrs)^10^	-0.22 (0.15)	0.10 (0.16)	0.21 (0.17)	-0.43 [-0.87, 0.02]	0.06^	-0.11 [-0.57, 0.35]		0.64
Total Week (hrs)^11^	-0.04 (0.09)	0.16 (0.09)^	0.52 (0.08)*	-0.56 [-0.80, -0.31]	< 0.001*	-0.36 [-0.60, -0.11]		0.004*
**Sleep Time**								
Weekday (hrs)^12^	0.28 (0.09)*	0.22 (0.09)*	0.03 (0.08)	0.25 [0.01, 0.48]	0.04*	0.19 [-0.04, 0.42]		0.11
Weekend (hrs)^13^	0.23 (0.18)	0.10 (0.19)	-0.13 (0.20)	0.36 [-0.16, 0.88]	0.18	0.22 [-0.31, 0.76]		0.41
Total Week (hrs)^14^	0.25 (0.10)*	0.19 (0.10)*	-0.10 (0.09)	0.35 [0.09, 0.60]	0.009*	0.30 [0.04, 0.55]		0.03*

^ 0.05 <  = *p* < 0.1

* *p* < 0.05

^1^ All models take into account the correlations between multiple measures from the same child and multiple children from the same center and adjust for treatment, time, treatment × time, center size, and outcome-specific significant confounding variables as noted below

^2^ Based on a linear mixed effects model of 455 observations (average observations per child = 1.5, average children per center = 37.9) adjusting for race/ethnicity; ICC = 0.47 for measures nested within children; ICC < 0.001 for children nested within centers

^3^ Based on a linear mixed effects model of 478 observations (average observations per child = 1.6, average children per center = 39.8) adjusting for baseline asthma and mother’s education; ICC =  < 0.001 for measures nested within children; ICC = 0.25 for children nested within centers

^4^ Based on a linear mixed effects model of 448 observations (average observations per child = 1.5, average children per center = 37.3) no additional adjustments; ICC = 0.08 for measures nested within children; ICC = 0.05 for children nested within centers

^5^ Based on a linear mixed effects model of 444 observations (average observations per child = 1.5, average children per center = 37.0) adjusting for asthma; ICC = 0.20 for measures nested within children; ICC =  < 0.001 for children nested within centers

^6^ Based on a linear mixed effects model of 461 observations (average observations per child = 1.6, average children per center = 38.4) adjusting for sex, mother’s education and language; ICC = 0.31 for measures nested within children; ICC < 0.001 for children nested within centers

^7^ Based on a linear mixed effects model of 470 observations (average observations per child = 1.6, average children per center = 39.2) adjusting for sex and ethnicity; ICC = 0.22 for measures nested within children; ICC = 0.001 for children nested within centers

^8^ Based on a linear mixed effects model of 479 observations (average observations per child = 1.6, average children per center = 39.9) adjusting for mother’s education and language; ICC = 0.36 for measures nested within children; ICC < 0.001 for children nested within centers

^9^ Based on a linear mixed effects model of 572 observations (average observations per child = 1.8, average children per center = 47.8) adjusting for sex, ethnicity and mother’s language; ICC = 0.42 for measures nested within children; ICC < 0.001 for children nested within centers

^10^ Based on a linear mixed effects model of 406 observations (average observations per child = 1.5, average children per center = 33.9) adjusting for sex and mother’s language; ICC = 0.37 for measures nested within children; ICC < 0.001 for children nested within centers

^11^ Based on a linear mixed effects model of 575 observations (average observations per child = 1.8, average children per center = 47.9) adjusting for sex, ethnicity and mother’s language; ICC = 0.47 for measures nested within children; ICC =  < 0.001 for children nested within centers

^12^ Based on a linear mixed effects model of 565 observations (average observations per child = 1.8, average children per center = 47.1) adjusting for ethnicity and mother’s language; ICC = 0.38 for measures nested within children; ICC = 0.01 for children nested within centers

^13^ Based on a linear mixed effects model of 421 observations (average observations per child = 1.5, average children per center = 34.1) adjusting for mother’s education and language; ICC = 0.16 for measures nested within children; ICC = 0.003 for children nested within centers

^14^ Based on a linear mixed effects model of 568 observations (average observations per child = 1.8, average children per center = 47.3) adjusting for mother’s language; ICC = 0.22 for measures nested within children; ICC = 0.008 for children nested within centers

There were no significant between-group differences for any diet outcome change score between control, CBI + HBI, or CBI, (Table 3). However, the directions of changes were in favor of children in CBI and CBI + HBI, except for fruit and vegetable intake among CBI children. However, there were significant within-group decreases in intake of total added sugar (-1.37 [95% CI: -2.55, -0.20] tsp, *p* = 0.022) and added sugar from beverages (-0.67 [-1.26, -0.07] tsp, *p* = 0.029) from baseline to post-intervention among children in the CBI. For children in the CBI + HBI, increases in vegetable (+ 0.31 [0.01, 0.61] cups, *p* = 0.043) and fruit and vegetable intake (+ 0.67 [0.06, 1.27] cups, *p* = 0.03) and a decrease in added sugar from beverages (-0.60 [-1.17, -0.02] tsp, *p* = 0.041) from baseline to post-intervention were found (Table 3).

Compared to control children (Table 3), weekday and total week screen time decreased significantly in both CBI + HBI (-0.57 [95% CI: -0.82, -0.33] hours and -0.56 [-0.80, -0.31] hours) and CBI groups (-0.43 [-0.68, -0.18] hours and -0.36 [-0.6, -0.11] hours). The difference in change in screen time from baseline to post-intervention between CBI + HBI and control groups approached significance for weekend screen time only (-0.43 [-0.87, 0.02] hours, *p* = 0.06). Within-group average weekday and total week screen time increased from baseline to post-intervention among children in CBI and control groups post-intervention. The overall trend for sleep time from baseline to post-intervention was similar, with children in both CBI + HBI and CBI groups increasing their weekday and total week sleep time, and sleep time among control children remaining the same or decreasing. Among CBI + HBI participants, there were significant increases in the change for weekday (0.25 [0.01, 0.48] hours, *p* = 0.04) and total week sleep time (0.35 [0.09, 0.60] hours, *p* = 0.009), and the increase in total week sleep time (0.30 [0.04, 0.55] hours, *p* = 0.03) reached significance in CBI children, compared to children in the control group.

## Discussion

¡Míranos! targeted low-income Latino children’s healthy EBRBs with evidence-based Head Start center policies and staff practices, and culturally tailored strategies for parental engagement [26, 27, 39]. The findings from this randomized controlled trial provide further evidence of the efficacy of early childhood obesity interventions in childcare settings on children’s EBRBs. Following the 8-month intervention, CBI + HBI parents reported positive changes in children’s at-home sleep and screen time, with similar results, but to a lesser extent in CBI children, compared to the control group. However, the effects of the intervention on changes in children’s PA and dietary outcomes were limited.

These findings are consistent with the conclusions of a recent systematic review [40] regarding the mixed results in PA and nutrition outcomes based on accelerometry and direct observations during center time. The impact of the CBI on the reported changes of EBRBs at home may be attributed to embedded intervention features. For example, childcare providers’ modeling of healthy behaviors and communication with parents, can influence children’s behaviors, including healthy eating and PA [41, 42]. In the ¡Míranos! program, teachers were not allowed to drink sugar-sweetened drinks in the presence of the children but were encouraged to taste all food at meals with children, thus modeling fruit and vegetable intake. Furthermore, various center-based activities required parental involvement or communication. The in-school fruit and vegetable intake contests, presence of healthy non-sugar sweetened beverages, and modeling of sleep and screen time included parent involvement. Food tastings conducted at school with new fruits and vegetables were accompanied by recipes provided to parents and stickers worn home by children communicated their involvement in the food tastings (e.g., “I tried carrots today!”). In the combined CBI + HBI group, parental involvement was associated with additional improvements in screen time, sleep on weekdays, and sleep for the entire week, and to a lesser extent in fruit and vegetable intake and decreased added sugar from beverages [31, 43]. The culturally tailored HBI with parent training and the parent-peer educator delivery of obesity prevention education in CBI + HBI contributed to the high level of fidelity of ¡Míranos! (e.g., attendance in poster sessions and completion of home visits), in contrast to prior intervention efforts that have encountered barriers to engaging Latino parents [44, 45].

Family-focused interventions are efficacious in the prevention and management of childhood obesity in primary care settings [34, 46]. However, childhood obesity prevention initiatives involving families have been less effective and more challenging to implement in childcare settings, especially in low-income minority populations including Latino children [44, 45, 47]. Parent engagement and participation are critical to influencing children’s EBRBs at home in *¡Míranos!*. The HBI constituted knowledge transference of all *¡Míranos!*-focused behavioral outcomes through peer-led education sessions that were strengthened further in Head Start home visits to support behavior change with evidence-based strategies. Interventions with a parental component have shown better outcomes in PA, diet, and other non-anthropometric indices [48] as compared to those without parental involvement [23]. Of note, these strategies to engage the parents and families were built on the existing Head Start infrastructure and standard practice and could be scaled in other organized childcare settings.

Inadequate fruit and vegetable intake and excessive intake of added sugar are consistently documented in US children, including preschool-aged children. Approximately one-third of children aged 2–5 do not meet the fruit recommendation and 90–98% do not meet the vegetable recommendation [49]. Early childhood eating habits extend into adulthood and can contribute to long-term health effects. Head Start centers utilize the Child and Adult Care Food Program or the National School Lunch Program which mandates federal guidelines for nutritious meals and snacks. Because of these requirements, children who attend Head Start centers tend to have a higher quality diet and healthy eating habits as compared to non-Head Start preschoolers [50, 51]. Further improving the diet at home through parental involvement has the potential to significantly impact the current and future eating habits of children who attend Head Start. Children in the CBI + HBI group increased fruit intake by 0.35 cups, vegetable intake by 0.31 cups, and fruit and vegetable intake by 0.67 cups per day. A recent meta-analysis revealed multicomponent interventions increased both fruit and vegetable intake by 0.37 cups per day. Two out of the five studies contained parental components which specifically increased vegetable consumption by children [40, 52]. Children aged 2–5 have reported intakes of 1.2–1.4 cups of fruit and 0.56–0.66 cups of vegetables per day [49]. Increasing 2/3 cups of fruits and vegetables per day at home, as found in this study, may aid in reaching the 2–3 cups of fruits and vegetables per day recommended by the Dietary Guidelines for Americans 2020 for this age group.

Sugar intake in children is associated with excessive weight gain. Children aged 1–5 years old reported having intakes of added sugar per day of greater than 10% of daily total kilocalories from sugar [53, 54], which exceeds the World Health Organization and the Dietary Guidelines for Americans 2020 recommendations [55, 56]. Additionally, children in this age group report an average intake of 49 g per day (11.7 teaspoons) [53] which exceeds the American Heart Association recommendation of fewer than 6 tsp per day [57]. In this study, added sugar intake at home was assessed and does not account for food eaten at Head Start centers. However, menu regulations for Head Start restrict added sugar from beverages and foods, so we speculate it would likely be a minor contributor to added sugar intake. While both CBI + HBI (-0.89 tsp (3.74 g)) and CBI (-1.37 tsp (5.75 g)) reduced total added sugar from baseline to post-intervention, neither group significantly reduced total added sugar intake compared to the control.

Shorter sleep duration in preschool-aged children is associated with a risk of overweight/obesity [25]. Findings from *¡Míranos!* Indicated increased weekday sleep among CBI participants, and increased weekday and total week sleep time among CBI + HBI, ranging from an additional 0.25–0.35 h/day (15–21 min/day). Interventions that improved sleep in preschool-aged children have shown an increase of 0.75 h/day (45 min/day) among children in a targeted at-home parental intervention [36] or documented improvements in the number of children who slept at least 11 h [58], while another reported no change in sleep time [59]. As indicated by the *¡Míranos!* findings, improvements in sleep time as small as 15 min per day, can increase total sleep time per week by 105 min (1.75 h) and contribute to reaching the benchmark of 11 h per night among preschool-aged children.

Reducing screen time by 17 min per day in children under age 5 has been achieved through interventions in non-center settings [60]. In the current study, weekday screen time increased among all groups that is consistent with age-associated upward trends in screen time [61]. However, the increase in weekday screen time was smallest in the CBI + HBI group (0.02 h/day [1.2 min/day]) compared to CBI (0.16 h/day [9.6 min/day]) and control (0.59 h/day [35.4 min/day]). The difference in weekday screen time between CBI + HBI and control at post intervention was -0.57 h/day (34.2 min/day) and for CBI it was -0.43 h/day (25.8 min/day). Similar results were found in total week screen time for children in the CBI + HBI (-0.56 h/day, 33.6 min/day) and CBI (-0.36 h/day, 21.6 min/day) as compared to the control. Parents in both the CBI and CBI + HBI groups reported that at the end of the intervention, their children spent less than 2 h per day of screen time during the weekday, weekend, and entire week as compared to the control group which exceeded 2 h per day. Reduction in excessive screen time among children has been associated with sedentary behavior and risk for overweight/obesity [62, 63]. The outcomes of *¡Míranos!* with both screen time reduction and increased sleep time provide support for parental rule-setting strategies aimed at curtailing young children’s behaviors that may have long-lasting detrimental lifestyle effects [25, 64].

However, the muted impact on screen time and sleep during the weekend days demonstrates unmet challenges in families from low socioeconomic status and racial/ethnic minorities [65]. Tandon and colleagues reported low SES families had less access to play equipment and more restrictive rules associated with PA, but more access to screens and more family screen time [65]. As Njoroge noted, parental attitudes about children’s screen time may explain racial/ethnic disparities [66]. Parents from low educational and low-income families were less likely to believe they could limit their children’s screen time and keep them busy without TV and more likely to believe preschool-aged children will benefit from educational TV programs [66]. Furthermore, increased screen time contributes to reduced sleep time or sleep quality in children [67, 68].

The lack of robust *¡Míranos!* intervention effect on child PA and dietary outcomes highlights the challenges of efforts to modify the home environment for resource-dependent behaviors in low-income families [69]. Although the *¡Míranos!* intervention incorporated evidenced-based strategies to build parents’ efficacy and skills to modify children’s PA and diet, the program did not provide increased access to, or financial support for, PA opportunities and healthy eating choices in the home or community, making it difficult for the low-income population to benefit from policy and environment intervention [70, 71]. For example, children in low-income families have less access to play equipment at home and to safe play environments, and at the same time have higher levels of at-home—media devices conducive to sedentary activities [65, 72]. Therefore, it is imperative to consider proportionate universalism in promoting the most appropriate solutions to address the resource-intensive challenges that are not commonly offered in obesity prevention initiatives [73].

Future research should test the feasibility if an equity-based approach to obesity prevention would address the root causes of obesity by providing population-specific interventions (i.e., removing financial barriers) aimed at ensuring all families have a fair and just opportunity to engage in PA and healthy eating practices [74, 75].

A major strength of *¡Míranos!* is the high fidelity with which the multiple program components were implemented in both intervention groups. Other strengths include the incorporation of evidence-based strategies to train Head Start staff and parents in managing EBRBs guided by a sound theoretical framework [24]. Furthermore, *¡Míranos!* was tailored to address the cultural, linguistic, and organizational needs of Head Start and study participants. Communications and interactions with participating families incorporated culturally and linguistically appropriate content and materials. Finally, although designed as an obesity prevention intervention, the key *¡Míranos!* messages focused primarily on modifying EBRBs rather than weight reduction per se, thus avoiding stigma and victim blaming. [56].

The limitations of this study include participant parent-reported behaviors at home and the use of a dietary screener to collect dietary data. Measures of parent-reported behaviors at home including screen-time, sleep, and adult facilitated physical activity have been used in previous studies but not validated. Dietary screeners are short food frequency questionnaires designed to capture general dietary information aimed to the reduce respondent burden but limit the utility and interpretation of the dietary data. Dietary data are typically underreported, but some studies show that it can depend on the type of foods reported, with overreporting of *healthy foods* and underreporting can occur for *unhealthy foods* [76]. To minimize the impact of language and cultural barriers on non-English speaking Hispanic parents, survey and log questions were posed in both English and Spanish to aid Spanish-speaking parents. The COVID-19 pandemic resulted in the loss of Cohort 2 participants, reducing the study sample and potentially impacting the statistical power to detect an intervention effect at 8 months post-intervention. A further limitation was that Head Start staff and data collectors were not blinded to the study center conditions. Since multiple strategies were implemented to target each of the EBRBs in *¡Míranos!*, we were not able to discern their effectiveness in affecting the behavioral outcome. Future research should examine the unique contribution and scalability of each strategy specific to the targeted population.

## Conclusion

*¡Míranos!*, multiple-component obesity prevention in early childcare centers with a culturally tailored home intervention was effective at improving the regulation of screen time and sleep but lacked a robust effect in modifying dietary and PA behaviors at home. The findings from this study support the role of parent education and training in changing young children’s health behaviors. However, future studies should investigate equity-related contextual factors that either enhance or mitigate the impact of obesity prevention initiatives in health-disparity populations.

## Data Availability

The datasets used and/or analyzed during the current study are available from the corresponding author on reasonable request.

## Abbreviations

AIC: Akaike’s information criterion
BIC: Bayesian information criterion
BMI: Body mass index
CACFP: Child and Adult Care Food Program
CBI: Center-based intervention group
EBRB: Energy balance-related behaviors
HBI: Home-based intervention group
LMM: Linear mixed effects model
NHANES: National Health and Nutrition Examination Survey
PA: Physical activity
SBB: Sugar-sweetened beverages
